# Diseases of the Udder and Teats1Abstract of a paper read before the Iowa State Veterinary Medical Association.

**Published:** 1899-08

**Authors:** W. H. Austin

**Affiliations:** Newton, Iowa


### DISEASES OF THE UDDER AND TEATS.[1]

By W. H. Austin, V.S.,
NEWTON, IOWA.

Among the first questions asked one in dealing with diseased conditions of the udder and teats are: "Can you cure this trouble?" "Will the milk be all right?" Some state as a reason that they are using the milk for their family, while others do not care, and will milk on the ground a portion of stringy milk, and then the balance in a pail; some ignorant, others indifferent as to the dangers involved in its sale and use.

Among the causes may be cited improper milking, neglecting to milk at the proper time, traumatic injuries, and atmospheric conditions. Inflammatory conditions of the udder frequently terminate in changes in the mucous membrane, connective-tissue growths that in part or wholly close up the opening of one or more teats. These growths can be localized at some portion of the duct, and usually develop faster when the cow is dry, and are ofttimes first discovered when the cow becomes fresh. The flow of milk to the quarter affected continues, and the udder becomes distended. The use of a quill or knitting-needle only gives partial relief, and in a week or ten days a case of garget, or caked bag, is to be dealt with.

[1] Abstract of a paper read before the Iowa State Veterinary Medical Association.

In other cases stringy milk is reported, perhaps tinged with blood, more frequently seen in old cows, ofttimes leaving small enlargements in one or more quarters which microscopical examination may prove to be tubercular in character, and tumefaction of the udder in various parts may rapidly follow, and in from two to four months evidences of general tuberculosis become manifest.

*Treatment.* I have not tried the virtues of electricity recently recommended in one of our veterinary periodicals. In dealing with strictures due to fibrous growths in the teat, take a strong grip with the thumb and forefinger around the knot, and force an exploring needle through the growth, and empty the quarter of its contents. This should be used daily for a week or ten days. In some cases the lance is to be resorted to, or a large trocar and canula may be inserted to eliminate the milk and pus.

A few cases that have come under my notice are of interest:

Case I. A single cow kept by owner, the milk and butter being used by the family. One teat was almost occluded; a hard obstacle could be felt high up in the duct. On introducing the needle, and enlarging the opening, much benefit was derived. A month or two afterward the owner reported the cow as giving stringy milk, and swelling up in this quarter; the animal's general condition was excellent. About a year or fourteen months after I was called to see this cow, which had become the property of another; found a hard, inflamed quarter, general emaciation, loss of appetite; on exertion, breathing fast and coughing at times. I endeavored to persuade the owner to destroy her, but he seemed to think that the loss of cud was the chief trouble, and desired to force some bacon down her throat to restore this function. She bore every evidence of general as well as mammary tuberculosis.

Case II. Purchased by a stock-buyer; nearly full-bred, short-horn, seven years old, in splendid condition. One quarter of the udder and its teat seemed slightly smaller than the other. The seller attributed this to an accident, the teat having been trodden on by another cow. This teat presented a hard growth in the duct. The other three teats were said to be in a normal condition. The cow being dry at the time, the purchaser was surprised to find that when she became fresh there was but one teat where the milk flowed properly. Strictures due to new growths were found, on close examination, in all three, and the exploring needle was used for a time, until she became dry again, and was sold.

Case III. Cow, nine years old, with history of a rapid falling off in flesh and milk, the latter stringy at times; had been an excellent milker for a year; had milked hard from one quarter. On examination I suspected tuberculosis. The udder was doughy and hard, and stricture of one quarter present. The owner was advised not to use the milk. Placed her under stimulating treatment, but she died. Autopsy revealed tuberculosis of mammary glands, especially the quarter that milked hard; also, lesions in the lungs and mediastinal glands.

Case IV. Full-bred Jersey, used in dairy; always had one blind teat; when fresh udder became feverish, and a chain of hard excrescences could be outlined in the diseased quarter. On examination much difficulty arose in getting anything from the quarter; was stringy and streaked with blood when obtained through a milk siphon. Repeated use of siphon and treatment re-established the milk secretion in this gland, but there were recurring attacks of mammitis.

Case V. My attention to this case was first brought through an inquiry by an old lady as to the cause of their milk having so peculiar an odor, to which I responded that it was probably due to not keeping utensils, etc., thoroughly clean. Two days later I was called to the farm of the dairyman supplying this lady with milk, and found one of his cows, a very large milker formerly, but giving but little now, run down in flesh, weak and staggering, growing gradually worse for some time. Her milk was placed in with the rest. The udder was larger than normal, especially one-half of it; doughy in feeling; partial stricture in one quarter. Milked her out, when an offensive odor was noticed. She lived but a day or two afterward. Her condition fully explained the odor complained of, and yet the owner continued selling that milk to his customers.

It is surely time that all dairies should be inspected, and such laws invoked as will prevent the sale of milk from such animals, and to compel reluctant owners to have these animals tested with tuberculin, that these many cases of garget or mammitis that are tubercular in character may be eliminated as a source of great danger to the consumers of milk and its products.

